# Social support for postpartum women and associated factors including online support to reduce stress and depression amidst COVID-19: Results of an online survey in Thailand

**DOI:** 10.1371/journal.pone.0289250

**Published:** 2023-07-27

**Authors:** Soo Jung Kim, Yin Min Aye, Danipa Panyarachun, Seo Ah Hong, Yan-Shing Chang

**Affiliations:** 1 Department of Public Health Sciences, Seoul National University, Seoul, Republic of Korea; 2 ASEAN Institute for Health Development, Mahidol University, Nakhon Pathom, Thailand; 3 Faculty of Medical Technology, Mahidol University, Nakhon Pathom, Thailand; 4 Institute for Health and Society, Hanyang University, Seoul, Republic of Korea; 5 Florence Nightingale Faculty of Nursing, Midwifery & Palliative Care, King’s College London, London, United Kingdom; Ghana Health Service (GHS) / African Forum for Primary Health Care (AfroPHC) / Liverpool School of Tropical Medicine (LSTM), GHANA

## Abstract

**Background:**

Social support for postpartum women helps mothers to recover from childbirth and fosters healthy infant development. However, the impacts of reduced interpersonal interactions inflicted by the COVID-19 outbreak on available social support for postpartum women have received little attention. Therefore, this study aimed to examine the levels of social support provided to postpartum women and associated factors in Thailand during the COVID-19 pandemic.

**Methods:**

A cross-sectional study was conducted from July to October 2021 using an anonymous online questionnaire. The responses of 840 eligible women up to six months postpartum in Thailand were obtained. The maternity social support scale was used to measure social support. Multivariate logistic regression was used to analyse the factors associated with social support among postpartum women.

**Results:**

About 57% of women reported to receive high support. Women in the high social support group were more likely to be married (aOR:2.70; 95% CI:1.57–4.66), have a university education or above (1.88; 1.35–2.64), have an intended pregnancy (2.06; 1.34–3.16), good health (2.01; 1.44–2.81), good sleep quality (1.62; 1.14–2.31), receive counsel from peers or family (1.56; 1.13–2.16), and use internet or social media to reduce stress and depression (1.51; 1.08–2.11). Meanwhile, women in the high social support group were significantly less likely to feed complementary foods to infants within 24 hours of completing the survey (0.28; 0.15–0.52).

**Conclusions:**

The results of this study indicated that more than half of the women reported high support and illustrated the important role played by family, peers, and professionals as well as online and remote channels in providing postpartum informational and emotional support during the pandemic. Online platforms and remote support may be considered to provide social support to postpartum women during a pandemic such as COVID-19.

## Introduction

According to House [1], social support refers to the material and immaterial aspects found in social relationships, such as empathy, physical assistance, beneficial information, and feedback. When women recover from the childbirth experience and start nurturing their newborn over the postpartum period [2], social support aids women in their recovery, prevents possible mental disorders and stress after childbirth [3, 4], and contributes to positive parenting [5] and bonding between the women and their newborns [6].

Postpartum women have faced changes in the provision of maternal social support since the onset of the coronavirus disease (COVID-19), resulting in increased anxieties and worries [7]. Some studies reported that more support was provided by family members who spent more time at home due to home-working or restricted outdoor movements [8]. On the other hand, some women experienced reduced social support due to fewer visits from family and peers [9]. A rise in domestic conflict and violence involving postpartum women was reported in households during the pandemic [10]. Such conflicting results concerning the availability of social support for postpartum women during the pandemic period require further attention. To our knowledge, research relating to maternal social support during the COVID-19 pandemic conducted in China [8], Japan [11], and the United States (US) [12], tended to focus on the relationship between COVID-19 and postpartum depression or mental distress. Moreover, no studies had specifically focused on levels of social support in association with sources of support for postpartum women during the COVID-19 pandemic in low and middle-income countries (LMICs), including Thailand, where the pandemic had a greater effect on less stable national economies with limited healthcare professionals and equipment compared to high-income countries with better resources [13].

To better understand social support among postpartum mothers during the COVID-19 pandemic, it is critical to understand the associated factors, including support received, support needed, and sources of supportive information. Previous studies have shown that sociodemographic factors such as older infant age [14], marital status [15], low income [15], and less education [16] were associated with low maternal social support. Furthermore, it has been reported that high social support may improve sleep quality [17] and play a role in infant feeding practice [10]. Meanwhile, women’s approaches to acquiring the necessary health information and emotional support during the ongoing challenges of COVID-19 are critical to understanding the sources of social support for postpartum women. Social support can come from various sources such as family, peers, and healthcare providers [18], all of whom have been affected by the pandemic [9] due to local or national lockdown and preventive measures to slow the spread of COVID-19. Video calls and chats using popular mobile applications such as LINE and Facebook have largely replaced family gatherings [12]. In the absence of in-person support gatherings of postpartum women, online social networking sites and groups have offered women a place to access information and social support [9]. In addition, while women would have been likely to need more informational support regarding the physical and emotional health of both themselves and their infants during the pandemic, their contacts with healthcare providers to receive professional advice in person might be reduced due to social distancing measures, decreased maternity facility utilization, and reducing physical interactions [10]. To bridge the research gap, this study planned to provide evidence on perceived social support levels and sources of informational and emotional support for postpartum women during the COVID-19 pandemic period.

This cross-sectional study aimed to examine the levels of maternal social support and associated factors among mothers up to six months postpartum amidst the COVID-19 pandemic in Thailand. In response to the limited information on maternal social support during the COVID-19 pandemic, our findings may help to raise awareness regarding social support and maternal health for postpartum women during pandemics such as COVID-19. Furthermore, the findings of this study may add to the achievement of the national target for the Sustainable Development Goals in relation to good health and well-being as well as poverty, and specifically, the expansion of social protection for the poor and vulnerable by 2030 [19].

## Materials and methods

### Study design and setting

A web-based cross-sectional study was conducted among postpartum women in Thailand from July to October 2021. Data were collected using an online Google Form. The inclusion criteria were postpartum women between the ages of 18–49, up to six months after childbirth, with online survey accessibility, and literate in the Thai language. Mothers not residing in Thailand during the survey period and with the inability to read the questions or have online access were excluded. Since this study used a convenience sampling via online, there was no sample size calculation. A total of 919 women completed the survey, with 840 of whom meeting the eligibility criteria. Based on the prevalence of social support, the sample was considered sufficient for this analysis.

Due to the social distancing measures imposed by local governments to combat the spread of COVID-19, convenience sampling was employed to recruit women. Study advertisements were distributed to postnatal clinics, midwifery/obstetric groups, and mother-baby groups, who were encouraged to share the study with others. Online advertisements were also employed via e-mail and popular local social media such as LINE and Facebook. The invitation included a link to the survey with an online written consent form providing information on the study purpose, procedure, voluntary nature, and confidentiality.

The study was approved by a Mahidol University Ethical Committee (No:2021/03-042). Respondents were informed that their participation was voluntary and provided with a digital consent form at the beginning of the survey. Informed consent was obtained by answering ‘yes’ to a consent question before they could access the questionnaire and was recorded digitally on the survey platform. Survey responses were anonymous while participants were asked provide contact details if they agreed to be approached for a follow-up survey.

### Measurement of variables

This anonymous and self-administered questionnaire for data collection was developed in English, translated into Thai, and translated back into English to ensure accuracy of translation. The dependent variable of maternal social support was assessed using the maternity social support scale (MSSS) to evaluate the levels of social support perceived by perinatal women. The assessment was simple, useful, time-efficient, objective, and easily interpreted [20]. The questionnaire consisted of six items to be ranked by participants according to a 5-point Likert-type scale, indicating their level of agreement or disagreement, with a possible total score ranging from 0–30. It was designed to assess the participants’ perceptions of the following: i) supportive friends; i) supportive family; iii) supportive husband or partner; iv) conflict with husband/partner; v) feeling controlled by husband/partner; and vi) feeling loved by husband/partner. Based on the results of the original study [20], cut-off points for MSSS were established; less than 19 (low support), 19–24 (medium support), and more than 24 (high support). In our study, due to the low number (n = 46, 5.48%) of respondents scoring within the 0–18 range, those scoring 24 or below were categorized into a low support group for further analysis.

Sociodemographic variables included the infant’s age and sex, mother’s age, occupation and education, monthly income, marital status, pregnancy intention, and mode of childbirth. Perceived changes in food security before and during the outbreak of the pandemic were also addressed with two questions: “Did you ever run out of food before the end of the month or cut down on the amount you ate to feed others in 2019 before the pandemic?” and comparably “Did you ever run out of food before the end of the month or cut down on the amount you ate to feed others in 2020/2021 during the COVID-19?”. The two questions were combined and categorized into i) insecure to insecure, ii) secure to insecure, iii) insecure to secure, and (iv) secure to secure. Variables relating to a COVID-19 positive diagnosis (yes or no), and COVID-19 vaccination uptake (yes or no) were also included. Questions concerning infant feeding practices in the last 24 hours, such as infant formula, breastfeeding, and any solid, semi-solid, or soft foods (including juice) were included. The women were also asked to mark the type of support provided for infant feeding and the specific support needed during the COVID-19 pandemic. Participants were also asked to rate their perception of self-health in terms of daily energy, overall physical well-being, and sleep quality of respondents over the previous 30 days before the survey completion date.

To examine sources of information, participants were asked about their sources of information on the COVID-19 pandemic, including the most frequently contacted sources as well as the most trusted. Mothers were asked if they had ever obtained information on postpartum depressive symptoms or depression. Participants were also asked to mark all the sources they had accessed concerning postpartum depressive symptoms or depression, and the forms of support or treatment used to reduce stress and depression during the postnatal period.

#### Data analysis

Statistical analysis was performed using SAS 9.3 (SAS Institute Inc., Cary, NC, USA). Descriptive statistics were used for frequency and percentages for categorical variables. The bivariate associations between the dependent variable (low/middle and high social support) and independent factors were assessed using the Chi-square test. Those independent variables with a p-value < 0.15 in the bivariate analyses were employed in the final multiple logistic regression to identify the predictors and strength of association with high social support. Statistical significance was considered with a p-value <0.05.

#### Inclusivity in global research

Additional information regarding the ethical, cultural, and scientific considerations specific to inclusivity in global research is included in the S1 Checklist.

## Results

Of the eligible respondents (n = 840), the majority were aged under 39 years, married, and mothers of infants aged three months or younger (Table 1). In bivariate associations, women with high social support were more likely to be younger (less than 39 years old), have a younger child (1–3 months old), a higher education, and live with a spouse (p<0.001), compared to their counterparts. They were also more likely to be on maternity leave, self-employed, or involve in a family-based business, have an intended pregnancy, and a caesarean section as the mode of childbirth (p<0.001). In addition, they also reported a relatively high socioeconomic status, such as having a high family income, and experienced better or no change in food security during the COVID-19 pandemic (p<0.01).

**Table 1:** Sociodemographic factors by social support levels among postpartum women in Thailand.

	Total		Social support				
			Low/Middle (0–24)		High (25–30)		
	n	(%)	n	(%)	n	(%)	p-value
Total number	840	(100.0)	362	(43.1)	478	(56.0)	
Infant’s age							
1–3 months	695	(82.7)	277	(76.5)	418	(87.5)	< .0001
4–6 months	145	(17.3)	85	(23.5)	60	(12.6)	
Infant’s sex (male)	435	(51.8)	179	(49.5)	256	(53.6)	0.2379
Mother’s age (years)							
18–29	489	(58.2)	230	(83.5)	259	(54.2)	0.001
30–39	318	(37.9)	113	(31.2)	205	(42.9)	
40–49	33	(3.9)	19	(5.3)	14	(2.9)	
Education of mother							
Secondary school or lower	458	(54.5)	243	(67.1)	215	(45.0)	< .0001
University or higher	382	(45.5)	119	(32.9)	263	(55.0)	
Marital status							
Married	759	(90.4)	305	(84.3)	454	(95.0)	< .0001
Single/divorced/widowed/separated	81	(9.6)	57	(15.8)	24	(5.0)	
Employment status							
Waged/employed	252	(30.0)	114	(31.5)	138	(28.9)	< .0001
Self-employed/family-based business	105	(12.5)	39	(10.8)	66	(13.8)	
On maternity leave	171	(20.4)	47	(13.0)	124	(25.9)	
Housewife/unemployed	312	(37.1)	162	(44.8)	150	(31.4)	
Residence (urban)	382	(45.5)	157	(43.4)	225	(47.1)	0.2861
Intended pregnancy (yes)	705	(83.9)	281.0	(77.6)	424	(88.7)	< .0001
Number of children	495	(58.9)	220	(60.8)	275	(57.5)	0.3442
Delivery mode (cesarean section)	351	(41.8)	126	(34.8)	225	(47.1)	0.0004
Low birth weight (<2.5 kg)	81	(9.6)	33	(9.1)	48	(10.0)	0.6526
Family income during COVID-19 pandemic †							
1st tertile (low)	278	(33.3)	136	(37.7)	142	(29.9)	0.0043
2nd tertile	309	(37.0)	138	(38.2)	171	(36.0)	
3rd tertile (high)	249	(29.8)	87	(24.1)	162	(34.1)	
Food insecurity status before and during COVID-19 pandemic							
No change (insecure–insecure)	236	(28.1)	123.0	(34.0)	113	(23.6)	0.0001
Worse (secure–insecure)	180	(21.4)	86.0	(23.8)	94	(19.7)	
Better or no change (secure–secure)	424	(50.5)	153.0	(42.3)	271	(56.7)	
Ever been diagnosed as COVID-19 positive (yes)	143	(17.0)	65.0	(18.0)	78	(16.3)	0.532

† missing values (n = 4): Missing values were excluded (not counted) in both the descriptive statistics and chi-squared tests

In Table 2, approximately 55–65% of women in the high social support group reported good health and sleep quality compared to their counterparts (p<0.0001). About 90% of women were breastfeeding, while infant formula and complementary feeding accounted for about 40% and 8%, respectively. Concerning social support groups, while no significant associations were reported with breastfeeding and infant formula feeding practice, a higher proportion of women with low support reported complementary feeding (p<0.0001). Regarding the infant feeding support received by women, while there was no significant difference in “no support” and “support from a healthcare professional” between groups, those in the high support group were more likely to receive help from a spouse/partner, friends or relatives (p<0.05) and online support groups (p<0.01). The majority of women reported they needed health care/medical support the most (76.4%), followed by financial support (51.2%) and mental health/emotional support (39%) during the COVID-19 pandemic, although no significant difference was observed between women with high and low social support.

**Table 2:** Health, sleep quality, infant feeding practice, and type of support by social support levels among postpartum women in Thailand.

	Total		Social support				
			Low/Middle (0–24)		High (25–30)		
	n	(%)	n	(%)	n	(%)	p-value
**Health-related factors**							
Self-reported overall health (good/excellent)	461	(54.9)	144	(40.0)	317	(66.6)	< .0001
Self-reported overall sleep quality (fairly/very good)	551	(65.6)	206	(57.1)	345	(72.3)	< .0001
Health problems during postpartum (yes)	73	(8.7)	39	(10.8)	34	(7.1)	0.0622
**Infant feeding practice last 24 hours**							
Breastfeeding (either at breast or using expressed milk)	750	(89.3)	326	(90.1)	424	(88.7)	0.5303
Infant formula	328	(39.0)	144	(39.8)	184	(38.5)	0.7053
Complementary foods	66	(7.9)	51	(14.1)	15	(3.1)	< .0001
**Support received for postnatal infant feeding** (mark all that apply)							
I receive no support for infant feeding	55	(6.6)	29	(8.0)	26	(5.4)	0.1357
From healthcare professionals	725	(86.3)	305	(84.3)	420	(87.9)	0.1315
From spouse/partner, friend, or relative	307	(36.6)	116	(32.0)	191	(40.0)	0.0183
From online support group (e.g., Facebook)	298	(35.5)	109	(30.1)	189	(39.5)	0.0047
**Type of support needed** specifically during the COVID-19 pandemic (mark all that apply)							
Health care/medical support	642	(76.4)	266	(73.5)	376	(78.7)	0.0798
Financial support	430	(51.2)	193	(53.3)	237	(49.6)	0.2837
Food support	275	(32.7)	121	(33.4)	154	(32.2)	0.7118
Mental health/emotional support	330	(39.3)	146	(40.3)	184	(38.5)	0.5891
Infant feeding support	269	(32.0)	116	(32.0)	153	(32.0)	0.9912

In terms of sources of information regarding COVID-19 (Table 3), over three-quarters of respondents chose social media such as Facebook or Instagram (76.7%), followed by the Internet (64.6%), mass media (60.1%), health professionals (52.6%), and family or personal networks (36.4% and 33.2%, respectively). However, they placed the most trust in hospitals, health centers, and health workers (46.6%). In the bivariate analyses, while there was no significant difference in obtaining information from family members, women with high social support were more likely to actively seek information from most sources (p<0.01). About half of the well-supported mothers considered hospitals and health workers to be the most trusted sources compared to their counterparts (39.2%) (p = 0.0025).

**Table 3:** Source of information according to social support level among postpartum women in Thailand.

	Total		Social support				
			Low/Middle (0–24)		High (25–30)		
	n	(%)	n	(%)	n	(%)	p-value
**Source of information on COVID-19**							
Source of information regarding the COVID-19 (mark all that apply)							
Family members	279	(33.2)	115	(31.8)	164	(34.3)	0.4386
Friends or neighbors or town/village leaders	306	(36.4)	108	(29.8)	198	(41.4)	0.0005
Hospitals, health centers, health workers	442	(52.6)	169	(46.7)	273	(57.1)	0.0027
Social media (Facebook, Instagram, etc.)	644	(76.7)	257	(71.0)	387	(81.0)	0.0007
Internet (Google, etc.)	543	(64.6)	212	(58.6)	331	(69.3)	0.0013
Mass media (TV, radio, newspaper)	505	(60.1)	191	(52.8)	314	(65.7)	0.0002
Source of information/advice on COVID-19 trusted the most							
Family members	27	(3.2)	17	(4.7)	10	(2.1)	0.0025
Friends or neighbors or town/village leaders	21	(2.5)	13	(3.6)	8	(1.7)	
Hospitals, health centers, health workers	391	(46.6)	142	(39.2)	249	(52.2)	
Social media (Facebook, Instagram, etc.)	137	(16.3)	65	(18.0)	72	(15.1)	
Internet (Google, etc.)	64	(7.6)	33	(9.1)	31	(6.5)	
Mass media (TV, radio, newspapers)	199	(23.7)	92	(25.4)	107	(22.4)	
**Information on postpartum depressive symptoms**							
Ever heard about postpartum depressive symptoms or depression?							
Yes	632	(75.2)	231	(63.8)	401	(83.9)	< .0001
No/not sure	208	(24.8)	131	(36.2)	77	(16.1)	
Sources accessed (mark all that apply)							
Internet/social media/Google	606	(72.1)	229	(63.3)	377	(78.9)	<0.0001
Doctors/healthcare staff	348	(41.4)	145	(40.1)	203	(42.5)	0.4820
Family/friends	214	(25.5)	81	(22.4)	133	(27.8)	0.0727
Forms of support/treatment to reduce stress and depression (mark all that apply)							
No support/treatment sought	149	(17.7)	69	(19.1)	80	(16.7)	0.3825
Counseling with peers/family	413	(49.2)	151	(41.7)	262	(54.8)	0.0002
Counseling/therapy with health professional	286	(34.1)	121	(33.4)	165	(34.5)	0.7405
Spiritual/faith-based support	117	(13.9)	47	(13.0)	70	(16.6)	0.4911
Physical exercise	124	(14.8)	40	(11.1)	84	(17.6)	0.0083
Internet/social media use	423	(50.4)	153	(42.3)	270	(56.5)	<0.0001

Regarding the source of information on postpartum depressive symptoms or depression (Table 3), about 75% of mothers reported having heard of the term “postpartum depressive symptoms or depression” and the most common source women accessed was social media (Internet, social media/Google) (72.1%). Among forms of support/treatment for reducing stress and depression, internet/social media were accessed the most (50.4%), followed by counseling with peers/family (49.2%), counseling/therapy with a health professional (34.1%), spiritual/faith-based support (13.9%), and physical exercise (14.8%). In the bivariate analyses with social support, women with high social support were more likely to have heard of the term (p<0.0001), while the internet/social media/Google were the most accessed sources (p<0.0001). In order to reduce stress and depression, women with high support were more likely to counsel peers or family (p<0.001), take physical exercise (p<0.05), and use internet/social media (p<0.0001) compared to their counterparts.

Variables with a p-value of less than 0.15 from bivariate analyses were employed to determine the factors of high social support in a multivariable logistic regression model (Table 4). Women in the high social support group were significantly more likely to be married (adjusted odds ratio (aOR) = 2.70; 95% confidence interval (CI) = 1.57–4.66), have an intended pregnancy (aOR = 2.06; 95% CI = 1.34–3.16), good or excellent health (aOR = 2.01; 95% CI = 1.44–2.81), a university education or above (aOR = 1.88; 95%CI = 1.35–2.64), good sleep quality (aOR = 1.62; 95% CI = 1.14–2.31), counsel with peers or family (aOR = 1.56; 95% CI = 1.13–2.16), and use the internet/social media for reduce stressing and depression (aOR = 1.51; 95% CI = 1.08–2.11). Meanwhile, women in the high social support group were less likely to feed complementary foods to infants within 24 hours of completing the survey (aOR = 0.28; 95% CI = 0.15–0.52).

**Table 4:** Adjusted odds ratios (AOR) and their 95% confidence intervals (CI) of high social support among postpartum women in Thailand.

	High Social Support		
	AOR	(95% CI)	
Education			
University+ vs. Secondary -	1.88	(1.35-	2.64)
Marital status			
Married vs. Other	2.70	(1.57-	4.66)
Intended pregnancy			
Yes vs. No	2.06	(1.34-	3.16)
Self-reported overall health			
Good/excellent vs. Fair/poor/very poor	2.01	(1.44-	2.81)
Self-reported overall sleep quality			
Fairly good/very good vs. Fairly bad/very bad	1.62	(1.14-	2.31)
Complementary foods in the last 24 hours			
Yes vs. No	0.28	(0.15-	0.52)
Counseling with peers/family to reduce stress and depression during the postnatal period			
Yes vs. No	1.56	(1.13-	2.16)
Internet/social media use to reduce stress and depression during the postnatal period			
Yes vs. No	1.51	(1.08-	2.11)

Variables with p-value <0.15 are included and Stepwise methods used to select the significant variables.

Hosmer and Lemeshow Goodness-of-Fit = 2.2252 and p = 0.9733

## Discussion

Although the findings cannot be generalized due to the use of convenience sampling, this study provides good insight into postpartum women’s experiences with social support during the COVID-19 pandemic in Thailand. Classified according to the cut-off points for MSSS [20], 56.9% of women in our study received high support, while 5.5% and 37.6% received low and medium support, respectively. These figures were higher than those presented in a study among mothers at up to 12 months postpartum in North West Ethiopia, where 53% reported medium social support, while 12.4% received high using the MSSS [21]. The high social support level in our study may be explained by many factors, such as cultural differences and the infant’s age. As shown in a Canadian study, women and infants received most social support immediately after childbirth [14]. Sharing some cultural postnatal practices with China and other Asian countries, Thai women are strongly advised to rest for the first few days, ranging from three days to a month at home after childbirth (Yuu-fai), with family or relatives often providing direct interpersonal care such as special food during this period [22]. A large number of the participants were women of infants between one and three months old (82.7%), and this group reported high social support.

Among several sociodemographic factors, women with a higher education were more likely to report high social support. High education leads to increased quality in social networks, which tend to provide larger amounts of material and immaterial resources [23] and better access to social support [24]. Thus, more attention should be given to postpartum women with low educational levels to help them better access various types of social support. Those with low education are also more likely to have experienced financial instability during the COVID-19 pandemic. Many women tend to work through the pregnancy and soon after the birth in Thailand [4]. In our study, 42.5% of postnatal women were either employed or self-employed, with 20.4% on maternity leave, illustrating that job security and a stable financial status are particularly relevant to Thai women in their childbearing years. The job security of many women has been affected by COVID-19, and financial strain can put extra pressure on them during the postpartum period [25]. About 50% of participants reported food insecurity during COVID-19 (28.1% reported constant food insecurity, and 21.4% felt insecure about food during COVID-19, despite having no such food insecurity beforehand). The majority of Thai women reported the need for financial support (51.2%), and one-third specifically required food support (32.7%) during the COVID-19 pandemic. Since more women in the low social support group experienced food insecurity, food and financial provision for low-income families should be emphasized, particularly during the pandemic period.

The extended Thai family traditionally provides a wide range of social support to perinatal women, although the family structure has significantly changed in recent years, and the COVID-19 pandemic resulted in further restrictions [26, 27]. Many studies have reported that relationships with a partner, family, and friends are associated with the emotional well-being of postpartum women [18, 28]. A study from the United Kingdom explained that a “reliable alliance,” notably with the woman’s partner and her parents, acted as the most important source of social support [29]. These findings were similar to those found in our study in that women cohabiting with a spouse or partner reported high social support, and several studies in Thailand showed that partners provided the most important physical and emotional resources to both postpartum women and newborns [29–31]. According to a study conducted in Japan, postpartum women with social support from others, such as extended family members or peers but without partner support were identified as a risk group for postpartum depression [5]. Such findings demonstrate the irreplaceable support of the partner. A Chinese study showed that interpersonal relationships, such as marital satisfaction and perceived caring from the mother-in-law, played important roles in sleep quality through social support [17]. Our study may support this by showing a positive association between self-reported physical health and sleep quality, and social support level. Since more physical hours are available due to high social support, the sleep quality and self-observed health satisfaction of mothers may increase as well [3], which is consistent with our study findings. In addition, a healthy partner relationship tends to promote communication over the family plan. Unmarried couples are less likely to plan for pregnancy, and unplanned pregnancy may result in emotional distress and potential depression [32]. Women with many social support resources through marriage, such as an extended family and a healthy relationship with their husband, may also plan for pregnancy more willingly, leading to better access to social support and, therefore, positive mental and physical health for mothers. On the other hand, during the pandemic period, an increase in domestic violence was reported, causing distress for perinatal women [10], and this calls for the education and promotion of healthy marital relationships.

Meanwhile, some studies have focused on the important roles of peers in the maternal well-being, stress, and depression of mothers during the pandemic. A Japanese study during the COVID-19 pandemic suggested that despite no significant family association, support from friends and others was strongly linked with the emotional status of postpartum women [11]. According to Thoits [33], social support provided by peers with the same stressors can be particularly helpful. Women experiencing similar challenges in the postpartum period may be able to support each other effectively. In addition, support for postnatal infant feeding from peers as well as the family may be linked to the provision of informational and physical support for postpartum women. Women preparing complementary foods may face physical and informational challenges since their preparation requires time, energy, and instruction. Experienced female relatives and friends could advise first-time mothers about feeding newborns, while partners and close relatives may also assist by preparing and feeding complementary foods to infants. A study in Western Kenya supported our finding that active participation by the infant’s father and grandmothers in providing physical support for infant feeding improved the woman’s perception of available support and was significantly associated with the intervention of grandmothers in dietary diversity for infants [34]. Another form of peer support comes from online social forums for perinatal women, which have largely replaced offline gatherings worldwide due to the COVID-19-related restrictions imposed by governments. Studies from the US, China, and Myanmar have discussed the virtues of online social networks, such as anonymity, social learning, accessibility, and provision of emotional and informational support for postpartum women [2, 18, 32]. A US study found that video calls with peers to be particularly helpful for promoting the well-being of postpartum women during the COVID-19 pandemic [12]. On the other hand, a cross-national study reported that information-seeking behavior online had a negative dose-response relationship with women’s perinatal mental health during the pandemic [7]. Such conflicting results warrant further research.

Despite the limited access to healthcare facilities and physical interaction with healthcare providers during the pandemic, they remained the most trusted source of information and advice. Consistent with a Chinese study [18], only half of our respondents received information regarding COVID-19 (52.6%) and postpartum depressive symptoms (41.4%) from health professionals. Our respondents perceived an acute lack of medical support from professionals who could have provided reliable health-related information. Internet and social media platforms such as Facebook and Line groups were the most frequently accessed source of information throughout the world, including in Thailand, even before the pandemic. Studies in Myanmar and Australia reported that social media and the internet appeal to perinatal women as a source of information to address potential threats and aid preparation during and after pregnancy, including the pandemic period [9, 32]. Further studies are warranted to examine the potential usage and benefit of online forums and mobile apps to supply informational and emotional support to postpartum women during future pandemics.

Interventions aiming to improve the physical and emotional status of postpartum women should address various ways of providing them with social support. When postpartum women were asked to choose the type of support they needed specifically during the COVID-19 pandemic period, regardless of being in low or high social support groups, they required healthcare/medical support (76.4%) and financial support (51.2%). As shown in our study, while trusting hospitals, health centers, and health workers most as a source of advice, Thai postpartum women mostly sought information and support online. In Thailand, breastfeeding support through remote channels involving phone calls and text messages in addition to the online approach, such as direct chats and video conferences with healthcare professionals, has already been implemented. During the ongoing pandemic, informational and emotional support provided via online or remote access against mental distress during the postpartum period should be considered since this would further lead to an improvement in perceived social support for postpartum women. A study in Singapore demonstrated the effectiveness of a mobile health application called “Home-but not Alone” in delivering social support to postpartum women. Volunteers trained by a psychiatrist in social support skills provided help through phone calls or text messages. The experiment resulted in a statistically significant association between the intervention and the perceived social support level of postpartum women [35–37]. Policymakers in Thailand could proactively adopt the experiment by mobilizing and training community volunteers and peers. Since an increased risk of perinatal mental disorders in LMICs was found among socially deprived women due to their limited health knowledge and financial means [31], such a trial may benefit postpartum women at risk of mental distress through early detection and the provision of additional emotional social support through systematic interactions with professionals, and with trained peers either online or remotely even during a pandemic such as COVID-19. Furthermore, Thailand is being encouraged to share its experience and best practices through South-South cooperation, involving the exchange of resources, technology, and knowledge among developing countries [19, 38]. Thailand now has the opportunity to strengthen its social protection of postpartum women and further contribute to the improved maternal health of developing countries. Nonetheless, it should be borne in mind that the rapid spread of misinformation via social media platforms poses a threat to public health. As directed by the United Nations Development Program, governments across the world should “step up to lead the fight against a growing tide of false, inflammatory and misleading information that threatens to worsen the already severe impacts of the virus” [39]. This study, therefore, calls on healthcare professionals and related experts to take concerted action against misinformation to prevent the spread of prevarication. Such commitment and efforts may contribute to the better health of women and their children.

This study has a few limitations concerning the interpretation of results. We cannot establish causality since our findings come from a cross-sectional survey. A longitudinal study is required to estimate causality. In addition, since the study includes mothers with infants only up to six months old and internet accessibility using convenience sampling techniques, the findings might not be representative of all postpartum women in Thailand. Although participants had to confirmed that they met the eligibility criteria before accessing the survey, we were unable to check the authenticity of those who received our survey information online such as via Line and Facebook. Nevertheless, the majority of respondents were identified and invited at postnatal clinics and mother-baby groups.

Despite these limitations, this study has certain strengths and to our knowledge, is one of the few to focus on social support for postpartum women, specifically during the COVID-19 pandemic. Furthermore, our study uniquely addresses the socioeconomic and health behavior of postpartum mothers regarding the perceived social support available in LMICs such as Thailand during the COVID-19 pandemic. This study provides insight into the sociodemographic challenges and potential benefits of various supportive channels such as community-based online platforms or remote support for postpartum women, especially those in need of emotional social support during the pandemic period. The results of this study imply that postpartum women, especially those with an economically and socially vulnerable status, could benefit from increased informational, physical, and emotional social support from a partner, immediate and extended family, peers, and health professionals via online and offline interaction. The findings may lead to the possible consideration of hybrid online and remote channels of providing peer and family support and reliable medical advice from professionals. Postpartum women in LIMCs, including Thailand, with limited access to health information and possible underlying health conditions, may benefit greatly from such an online platform.

## Conclusion

Our results indicated that socially deprived women tended to receive a lower level of social support, highlighting the important role of family, peers, and professionals as well as online platforms and remote channels for accessing postpartum informational and emotional support during the pandemic. Well-developed online platforms and remote support may need to be considered to provide effective social support to postpartum women during a pandemic such as COVID-19.

## Supporting information

S1 Checklist Inclusivity in global research. (DOCX)
